# Supplementary material for: Excess-entropy scaling in supercooled binary mixtures
Source: Nat Commun. 2020 Aug 27;11:4300. doi: 10.1038/s41467-020-17948-1 (PMC7453028; doi:10.1038/s41467-020-17948-1)
Supplement: Supplementary file 1 — Supplementary information [file 41467_2020_17948_MOESM1_ESM.pdf]

Supplementary Information

"Excess-Entropy Scaling in Supercooled Binary Mixtures"

Bell et al.

Additional viscosity results. The figure below displays excess-entropy scaling for viscosity corresponding to Figs. 1c and d of the main text.

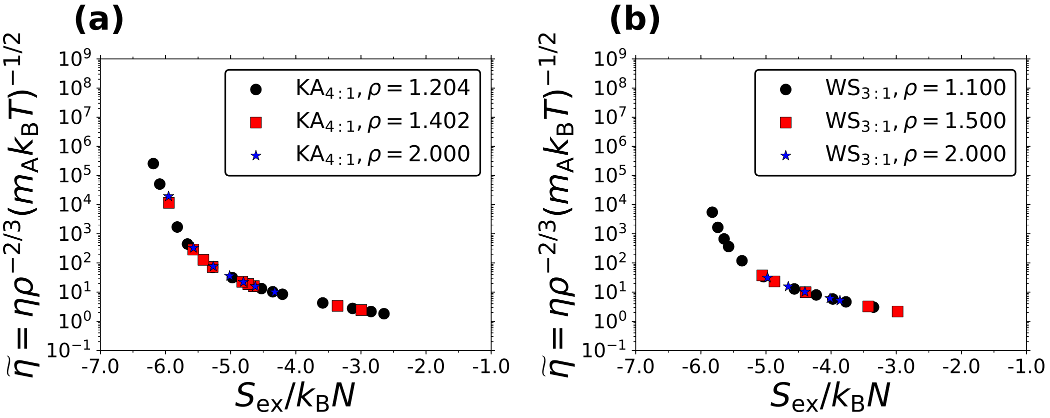

Supplementary Figure 1 Excess-entropy scaling for viscosity. **a** The 4:1 KA mixture at the densities: ρ = 1.204, 1.402, 2.000. **b** The 3:1 WS mixture at the densities: ρ = 1.100, 1.500, 2.000. An excellent collapse is found for both systems.

B-particle quasiuniversality. Results for the smaller B-particle are presented here where the main text focused on the larger A-particle. We find similar results as for the A-particle but with a bit larger scatter in the data.

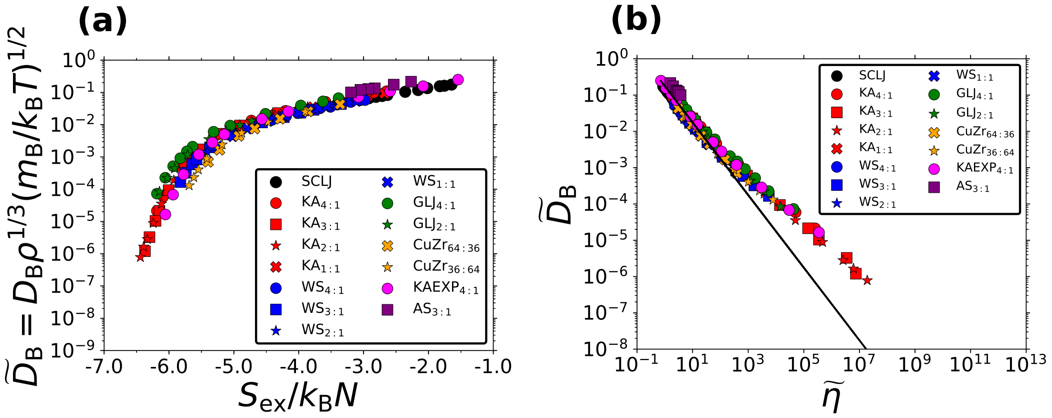

Supplementary Figure 2 B-particle diffusion coefficient results. **a** Reduced B-particle diffusion coefficients as a function of S_{ex} for all mixtures and compositions; a quasiuniversal curve is observed. **b** Reduced B-particle diffusion coefficients against reduced viscosities. The black line is SE with slope -1 fitted to 1:1 KA data.

Additional tests for quasiuniversal behavior. AB and BB-particle RDFs for the 2:1 and 4:1 KA mixtures are compared in Supplementary Figure 3 (see also Fig. 5 of the main text). Clear differences are noted for these two systems.

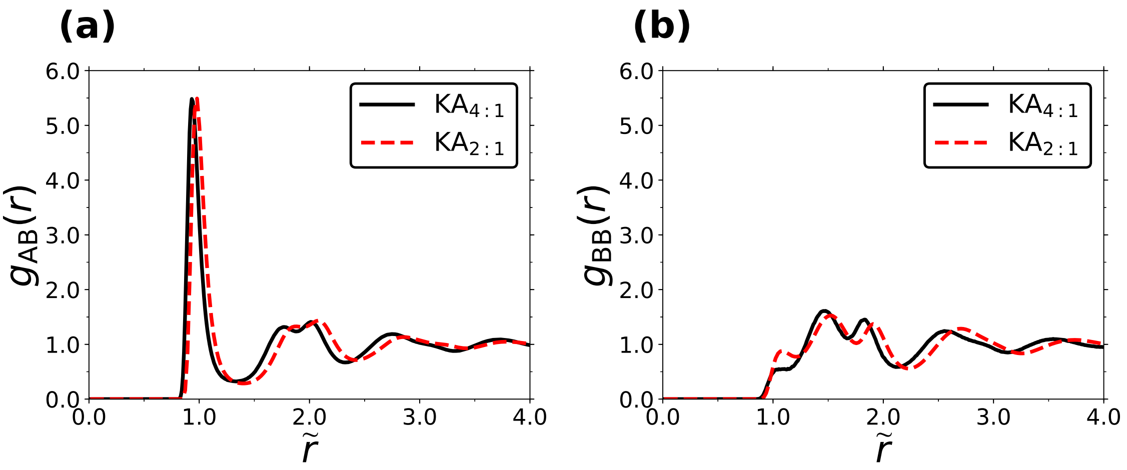

Supplementary Figure 3 Test of radial distribution function quasiuniversality where $\tilde{r} = \rho^{1/3} r$ and $\tilde{t} = t\rho^{1/3}(k_B T/m_A)^{1/2}$. a AB-particle RDFs for the KA mixture at two different compositions (4:1 and 2:1) for state points with almost identical S_{ex} and $\widetilde{D}_A$. For 4:1 KA: $\rho = 1.204$ and $T = 0.440$ and for 2:1 KA: $\rho = 1.400$ and $T = 0.550$. **b** BB-particle RDFs with details given in **a**.

Supplementary Figure 4 shows a comparison of the (reduced unit) Voronoi volume distribution for the same systems as the main text. We find also here differences between the compared systems, being most pronounced for the KA mixtures (Supplementary Figure 4a). The bimodal distribution is due to different particle sizes in the binary mixtures.

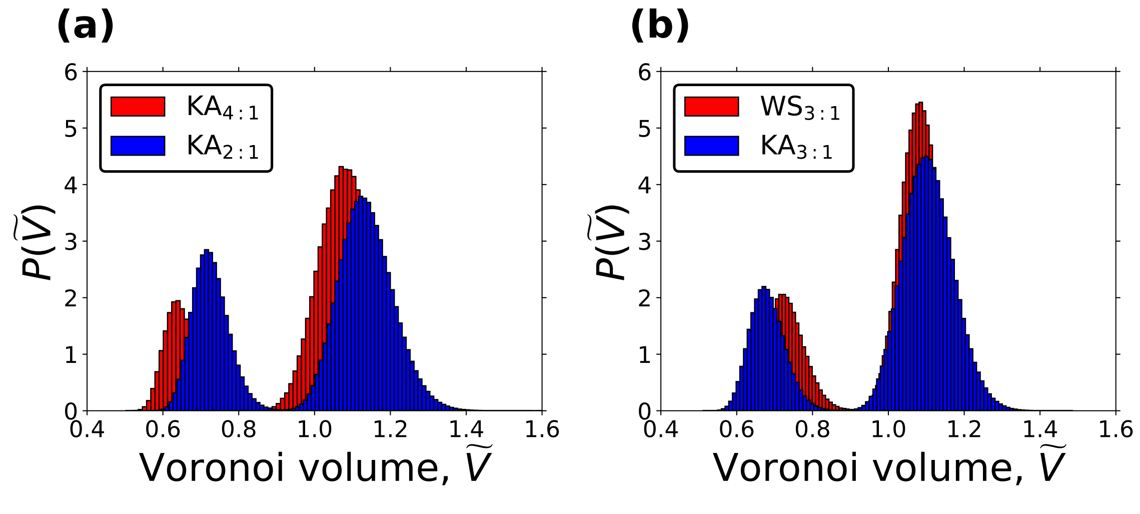

Supplementary Figure 4 Test of Voronoi volume quasiuniversality where $\widetilde{V} = \rho V$. a Voronoi volume distribution for the KA mixture at two different compositions (4:1 and 2:1) for state points with almost identical S_{ex} and $\widetilde{D}_A$. For 4:1 KA: $\rho = 1.204$ and $T = 0.440$ and for 2:1 KA: $\rho = 1.400$ and $T = 0.550$. **b** Voronoi volume distribution for the KA and WS mixtures at the same 3:1 composition for state points with almost identical S_{ex} and $\widetilde{D}_A$. For 3:1 KA: $\rho = 1.400$ and $T = 0.900$ and for 3:1 WS: $\rho = 1.100$ and $T = 0.415$.

Tables with simulation data.

Supplementary Table 1 Simulations for potentials in the "Lennard-Jones" unit system based upon the length scale and energy scale of the A particle, R is the U-W correlation, and γ is the density-scaling exponent

pot.	N_A	N_B	m_A	m_B	T	ρ	$S_\mathrm{ex}/(Nk_\mathrm{B})$	η	D_A	D_B	R	γ
AS	750	250	2.0	1.0	0.7	1.1000	-3.195	$4.663\times10^{+00}$	2.134×10^{-02}	8.117×10^{-02}	0.776	4.628
AS	750	250	2.0	1.0	0.8	1.1000	-3.048	$4.331\times10^{+00}$	2.568×10^{-02}	1.042×10^{-01}	0.818	4.824
AS	750	250	2.0	1.0	0.9	1.1000	-2.928	$4.119\times10^{+00}$	3.102×10^{-02}	1.175×10^{-01}	0.846	4.936
AS	750	250	2.0	1.0	1	1.1000	-2.826	$3.914\times10^{+00}$	3.591×10^{-02}	1.301×10^{-01}	0.867	5.010
AS	750	250	2.0	1.0	1.4	1.1000	-2.535	$3.559\times10^{+00}$	5.549×10^{-02}	2.003×10^{-01}	0.914	5.123
AS	750	250	2.0	1.0	2	1.1000	-2.268	$3.357\times10^{+00}$	8.352×10^{-02}	2.946×10^{-01}	0.945	5.127
GLJ	668	334	1.0	1.0	0.8	1.3500	-6.147	$1.574\times10^{+04}$	2.442×10^{-05}	6.816×10^{-05}	0.957	6.461
GLJ	668	334	1.0	1.0	0.85	1.3500	-6.025	$4.191\times10^{+03}$	7.500×10^{-05}	1.846×10^{-04}	0.959	6.416
GLJ	668	334	1.0	1.0	0.9	1.3500	-5.913	$1.387\times10^{+03}$	1.874×10^{-04}	3.758×10^{-04}	0.960	6.388
GLJ	668	334	1.0	1.0	1	1.3500	-5.713	$3.601\times10^{+02}$	5.950×10^{-04}	1.065×10^{-03}	0.964	6.343
GLJ	668	334	1.0	1.0	1.2	1.3500	-5.385	$9.751\times10^{+01}$	2.216×10^{-03}	3.496×10^{-03}	0.969	6.260
GLJ	668	334	1.0	1.0	1.4	1.3500	-5.124	$5.192\times10^{+01}$	4.536×10^{-03}	6.793×10^{-03}	0.972	6.190
GLJ	668	334	1.0	1.0	1.6	1.3500	-4.909	$3.573\times10^{+01}$	7.323×10^{-03}	1.112×10^{-02}	0.975	6.129
GLJ	668	334	1.0	1.0	1.8	1.3500	-4.728	$2.759\times10^{+01}$	1.045×10^{-02}	1.582×10^{-02}	0.978	6.076
GLJ	668	334	1.0	1.0	2	1.3500	-4.573	$2.299\times10^{+01}$	1.448×10^{-02}	1.993×10^{-02}	0.979	6.030
GLJ	800	200	1.0	1.0	0.75	1.2000	-6.187	$4.175\times10^{+04}$	1.229×10^{-05}	5.938×10^{-05}	0.956	6.236
GLJ	800	200	1.0	1.0	0.8	1.2000	-6.058	$5.480\times10^{+03}$	4.749×10^{-05}	1.906×10^{-04}	0.955	6.248
GLJ	800	200	1.0	1.0	0.85	1.2000	-5.936	$1.852\times10^{+03}$	1.349×10^{-04}	4.479×10^{-04}	0.960	6.229
GLJ	800	200	1.0	1.0	0.9	1.2000	-5.826	$8.170\times10^{+02}$	2.923×10^{-04}	8.186×10^{-04}	0.962	6.219
GLJ	800	200	1.0	1.0	0.95	1.2000	-5.725	$4.464\times10^{+02}$	5.292×10^{-04}	1.367×10^{-03}	0.964	6.197
GLJ	800	200	1.0	1.0	1	1.2000	-5.635	$2.476\times10^{+02}$	8.524×10^{-04}	1.961×10^{-03}	0.966	6.190
GLJ	800	200	1.0	1.0	1.2	1.2000	-5.312	$7.764\times10^{+01}$	2.825×10^{-03}	6.230×10^{-03}	0.971	6.135
GLJ	800	200	1.0	1.0	1.4	1.2000	-5.057	$4.205\times10^{+01}$	5.535×10^{-03}	1.044×10^{-02}	0.974	6.088
GLJ	800	200	1.0	1.0	2	1.2000	-4.512	$1.893\times10^{+01}$	1.674×10^{-02}	2.901×10^{-02}	0.981	5.964
GLJ	800	200	1.0	1.0	3	1.2000	-3.968	$1.185\times10^{+01}$	3.847×10^{-02}	6.415×10^{-02}	0.986	5.812
GLJ	800	200	1.0	1.0	4	1.2000	-3.627	$9.683\times10^{+00}$	6.272×10^{-02}	9.945×10^{-02}	0.989	5.703
GLJ	800	200	1.0	1.0	5	1.2000	-3.386	$8.580\times10^{+00}$	8.666×10^{-02}	1.398×10^{-01}	0.991	5.621
KA	5000	5000	1.0	1.0	0.75	1.4500	-4.676	$1.784\times10^{+01}$	6.784×10^{-03}	8.721×10^{-03}	0.952	5.438
KA	5000	5000	1.0	1.0	0.8	1.4500	-4.574	$1.564\times10^{+01}$	8.173×10^{-03}	1.045×10^{-02}	0.955	5.420
KA	5000	5000	1.0	1.0	0.9	1.4500	-4.394	$1.264\times10^{+01}$	1.134×10^{-02}	1.437×10^{-02}	0.959	5.362
KA	5000	5000	1.0	1.0	1	1.4500	-4.239	$1.111\times10^{+01}$	1.454×10^{-02}	1.828×10^{-02}	0.962	5.336
KA	5000	5000	1.0	1.0	1.2	1.4500	-3.984	$8.708\times10^{+00}$	2.148×10^{-02}	2.692×10^{-02}	0.967	5.244
KA	5000	5000	1.0	1.0	1.4	1.4500	-3.782	$7.562\times10^{+00}$	2.859×10^{-02}	3.637×10^{-02}	0.971	5.200
KA	5000	5000	1.0	1.0	1.6	1.4500	-3.616	$6.845\times10^{+00}$	3.653×10^{-02}	4.549×10^{-02}	0.974	5.154
KA	5000	5000	1.0	1.0	2	1.4500	-3.358	$5.913\times10^{+00}$	5.212×10^{-02}	6.539×10^{-02}	0.979	5.089
KA	5000	5000	1.0	1.0	3	1.4500	-2.940	$5.079\times10^{+00}$	9.299×10^{-02}	1.154×10^{-01}	0.984	4.953
KA	5000	5000	1.0	1.0	4	1.4500	-2.679	$4.765\times10^{+00}$	1.312×10^{-01}	1.641×10^{-01}	0.987	4.872
KA	6668	3334	1.0	1.0	0.48	1.4000	-6.441	$1.658\times10^{+07}$	1.112×10^{-07}	4.837×10^{-07}	0.959	5.202
KA	6668	3334	1.0	1.0	0.49	1.4000	-6.392	$5.362\times10^{+06}$	2.489×10^{-07}	1.031×10^{-06}	0.945	5.236
KA	6668	3334	1.0	1.0	0.5	1.4000	-6.334	$2.280\times10^{+06}$	4.702×10^{-07}	1.775×10^{-06}	0.948	5.168
KA	6668	3334	1.0	1.0	0.52	1.4000	-6.243	$4.259\times10^{+05}$	1.786×10^{-06}	5.734×10^{-06}	0.955	5.258
KA	6668	3334	1.0	1.0	0.55	1.4000	-6.117	$4.623\times10^{+04}$	8.850×10^{-06}	2.346×10^{-05}	0.958	5.244
KA	6668	3334	1.0	1.0	0.6	1.4000	-5.925	$3.624\times10^{+03}$	6.399×10^{-05}	1.316×10^{-04}	0.960	5.215
KA	6668	3334	1.0	1.0	0.7	1.4000	-5.602	$2.970\times10^{+02}$	5.580×10^{-04}	8.972×10^{-04}	0.965	5.180
KA	6668	3334	1.0	1.0	0.8	1.4000	-5.338	$9.441\times10^{+01}$	1.675×10^{-03}	2.446×10^{-03}	0.968	5.151
KA	6668	3334	1.0	1.0	1	1.4000	-4.927	$3.386\times10^{+01}$	5.237×10^{-03}	7.289×10^{-03}	0.973	5.097
KA	6668	3334	1.0	1.0	1.2	1.4000	-4.618	$2.012\times10^{+01}$	9.949×10^{-03}	1.358×10^{-02}	0.977	5.059
KA	6668	3334	1.0	1.0	1.4	1.4000	-4.373	$1.508\times10^{+01}$	1.520×10^{-02}	2.056×10^{-02}	0.979	5.008
KA	6668	3334	1.0	1.0	1.8	1.4000	-4.006	$1.082\times10^{+01}$	2.713×10^{-02}	3.643×10^{-02}	0.983	4.962
KA	6668	3334	1.0	1.0	2	1.4000	-3.862	$9.835\times10^{+00}$	3.334×10^{-02}	4.496×10^{-02}	0.984	4.937
KA	6668	3334	1.0	1.0	3	1.4000	-3.361	$7.271\times10^{+00}$	6.592×10^{-02}	8.908×10^{-02}	0.988	4.848
KA	6668	3334	1.0	1.0	4	1.4000	-3.052	$6.411\times10^{+00}$	1.001×10^{-01}	1.343×10^{-01}	0.991	4.783
KA	7500	2500	1.0	1.0	0.68	1.4000	-6.370	$7.694\times10^{+06}$	1.658×10^{-07}	8.841×10^{-07}	0.971	4.987
KA	7500	2500	1.0	1.0	0.7	1.4000	-6.301	$3.649\times10^{+06}$	5.207×10^{-07}	2.446×10^{-06}	0.973	4.898
KA	7500	2500	1.0	1.0	0.73	1.4000	-6.208	$3.723\times10^{+05}$	1.984×10^{-06}	7.956×10^{-06}	0.970	4.957

Continued on next page

Supplementary Table 1 Simulations for potentials in the "Lennard-Jones" unit system based upon the length scale and energy scale of the A particle, R is the U-W correlation, and γ is the density-scaling exponent

pot.	N_{A}	N_{B}	m_{A}	m_{B}	T	ρ	$S_{\mathrm{ex}}/(Nk_{\mathrm{B}})$	η	D_{A}	D_{B}	R	γ
KA	7500	2500	1.0	1.0	0.75	1.4000	-6.148	$1.541\times10^{+05}$	4.525×10^{-06}	1.655×10^{-05}	0.972	4.935
KA	7500	2500	1.0	1.0	0.8	1.4000	-6.006	$1.543\times10^{+04}$	2.666×10^{-05}	7.482×10^{-05}	0.976	4.937
KA	7500	2500	1.0	1.0	0.9	1.4000	-5.754	$1.078\times10^{+03}$	2.442×10^{-04}	5.140×10^{-04}	0.977	4.921
KA	7500	2500	1.0	1.0	1	1.4000	-5.540	$2.694\times10^{+02}$	8.438×10^{-04}	1.532×10^{-03}	0.979	4.913
KA	7500	2500	1.0	1.0	1.2	1.4000	-5.186	$7.380\times10^{+01}$	3.120×10^{-03}	5.112×10^{-03}	0.982	4.893
KA	7500	2500	1.0	1.0	1.4	1.4000	-4.906	$3.945\times10^{+01}$	6.482×10^{-03}	9.986×10^{-03}	0.984	4.873
KA	7500	2500	1.0	1.0	1.8	1.4000	-4.485	$2.061\times10^{+01}$	1.482×10^{-02}	2.219×10^{-02}	0.987	4.837
KA	7500	2500	1.0	1.0	2	1.4000	-4.320	$1.717\times10^{+01}$	1.954×10^{-02}	2.892×10^{-02}	0.988	4.821
KA	820	204	1.0	1.0	0.42	1.2040	-6.187	$1.878\times10^{+05}$	2.581×10^{-06}	1.299×10^{-05}	0.933	5.227
KA	820	204	1.0	1.0	0.44	1.2040	-6.089	$3.818\times10^{+04}$	9.044×10^{-06}	3.666×10^{-05}	0.931	5.160
KA	820	204	1.0	1.0	0.5	1.2040	-5.820	$1.369\times10^{+03}$	1.272×10^{-04}	3.369×10^{-04}	0.940	5.163
KA	820	204	1.0	1.0	0.54	1.2040	-5.663	$3.715\times10^{+02}$	3.515×10^{-04}	8.593×10^{-04}	0.944	5.162
KA	820	204	1.0	1.0	0.66	1.2040	-5.276	$6.542\times10^{+01}$	1.883×10^{-03}	3.546×10^{-03}	0.954	5.152
KA	820	204	1.0	1.0	0.78	1.2040	-4.974	$3.141\times10^{+01}$	4.296×10^{-03}	7.260×10^{-03}	0.961	5.137
KA	820	204	1.0	1.0	0.9	1.2040	-4.730	$2.094\times10^{+01}$	7.230×10^{-03}	1.199×10^{-02}	0.966	5.119
KA	820	204	1.0	1.0	1.02	1.2040	-4.527	$1.512\times10^{+01}$	1.054×10^{-02}	1.745×10^{-02}	0.969	5.101
KA	820	204	1.0	1.0	1.14	1.2040	-4.354	$1.243\times10^{+01}$	1.418×10^{-02}	2.284×10^{-02}	0.972	5.083
KA	820	204	1.0	1.0	1.26	1.2040	-4.205	$1.074\times10^{+01}$	1.810×10^{-02}	2.837×10^{-02}	0.975	5.064
KA	820	204	1.0	1.0	2	1.2040	-3.586	$6.822\times10^{+00}$	4.466×10^{-02}	6.807×10^{-02}	0.983	4.968
KA	820	204	1.0	1.0	3	1.2040	-3.128	$5.425\times10^{+00}$	8.278×10^{-02}	1.215×10^{-01}	0.988	4.878
KA	820	204	1.0	1.0	4	1.2040	-2.845	$4.887\times10^{+00}$	1.212×10^{-01}	1.835×10^{-01}	0.990	4.812
KA	820	204	1.0	1.0	5	1.2040	-2.646	$4.605\times10^{+00}$	1.638×10^{-01}	2.290×10^{-01}	0.992	4.764
KA	820	204	1.0	1.0	1	1.4016	-5.949	$1.445\times10^{+04}$	4.621×10^{-05}	1.407×10^{-04}	0.983	4.807
KA	820	204	1.0	1.0	1.2	1.4016	-5.576	$3.931\times10^{+02}$	7.404×10^{-04}	1.578×10^{-03}	0.985	4.795
KA	820	204	1.0	1.0	1.3	1.4016	-5.419	$1.821\times10^{+02}$	1.523×10^{-03}	2.891×10^{-03}	0.986	4.789
KA	820	204	1.0	1.0	1.4	1.4016	-5.277	$1.080\times10^{+02}$	2.504×10^{-03}	4.566×10^{-03}	0.987	4.784
KA	820	204	1.0	1.0	1.8	1.4016	-4.822	$3.782\times10^{+01}$	8.135×10^{-03}	1.416×10^{-02}	0.989	4.760
KA	820	204	1.0	1.0	1.9	1.4016	-4.730	$3.261\times10^{+01}$	1.004×10^{-02}	1.622×10^{-02}	0.990	4.753
KA	820	204	1.0	1.0	2	1.4016	-4.644	$2.841\times10^{+01}$	1.170×10^{-02}	1.957×10^{-02}	0.990	4.747
KA	820	204	1.0	1.0	5	1.4016	-3.359	$9.433\times10^{+00}$	8.301×10^{-02}	1.287×10^{-01}	0.995	4.621
KA	820	204	1.0	1.0	7	1.4016	-2.994	$8.054\times10^{+00}$	1.368×10^{-01}	2.012×10^{-01}	0.996	4.571
KA	820	204	1.0	1.0	5	2.0000	-5.953	$6.849\times10^{+04}$	5.946×10^{-05}	1.804×10^{-04}	0.998	4.338
KA	820	204	1.0	1.0	6	2.0000	-5.571	$1.278\times10^{+03}$	1.332×10^{-03}	2.765×10^{-03}	0.998	4.334
KA	820	204	1.0	1.0	7	2.0000	-5.267	$3.211\times10^{+02}$	4.848×10^{-03}	9.031×10^{-03}	0.998	4.328
KA	820	204	1.0	1.0	8	2.0000	-5.016	$1.607\times10^{+02}$	9.924×10^{-03}	1.780×10^{-02}	0.999	4.323
KA	820	204	1.0	1.0	9	2.0000	-4.804	$1.059\times10^{+02}$	1.633×10^{-02}	2.678×10^{-02}	0.999	4.318
KA	820	204	1.0	1.0	10	2.0000	-4.622	$7.916\times10^{+01}$	2.382×10^{-02}	3.756×10^{-02}	0.999	4.313
KA	820	204	1.0	1.0	12	2.0000	-4.323	$5.524\times10^{+01}$	3.966×10^{-02}	6.365×10^{-02}	0.999	4.304
KAEXP	820	204	1.0	1.0	4×10^{-06}	0.0010	-6.056	$7.053\times10^{+00}$	1.072×10^{-07}	3.298×10^{-07}	0.996	3.150
KAEXP	820	204	1.0	1.0	4.2×10^{-06}	0.0010	-5.937	6.108×10^{-01}	5.206×10^{-07}	1.390×10^{-06}	0.996	3.151
KAEXP	820	204	1.0	1.0	4.5×10^{-06}	0.0010	-5.776	6.409×10^{-02}	3.054×10^{-06}	6.083×10^{-06}	0.996	3.144
KAEXP	820	204	1.0	1.0	5×10^{-06}	0.0010	-5.541	8.472×10^{-03}	1.516×10^{-05}	2.655×10^{-05}	0.996	3.136
KAEXP	820	204	1.0	1.0	5.5×10^{-06}	0.0010	-5.331	2.571×10^{-03}	3.998×10^{-05}	6.575×10^{-05}	0.996	3.126
KAEXP	820	204	1.0	1.0	6×10^{-06}	0.0010	-5.146	1.333×10^{-03}	7.423×10^{-05}	1.227×10^{-04}	0.996	3.117
KAEXP	820	204	1.0	1.0	8×10^{-06}	0.0010	-4.568	4.218×10^{-04}	2.807×10^{-04}	4.254×10^{-04}	0.997	3.085
KAEXP	820	204	1.0	1.0	1×10^{-05}	0.0010	-4.159	2.586×10^{-04}	5.593×10^{-04}	8.130×10^{-04}	0.997	3.057
KAEXP	820	204	1.0	1.0	2×10^{-05}	0.0010	-3.081	1.168×10^{-04}	2.419×10^{-03}	3.222×10^{-03}	0.997	2.954
KAEXP	820	204	1.0	1.0	3×10^{-05}	0.0010	-2.590	9.363×10^{-05}	4.331×10^{-03}	5.973×10^{-03}	0.997	2.885
KAEXP	820	204	1.0	1.0	5×10^{-05}	0.0010	-2.081	7.824×10^{-05}	8.638×10^{-03}	1.105×10^{-02}	0.996	2.789
KAEXP	820	204	1.0	1.0	0.0001	0.0010	-1.543	7.218×10^{-05}	1.828×10^{-02}	2.492×10^{-02}	0.996	2.644
SCLJ	1024		1.0		0.7	0.8500	-3.671	$3.471\times10^{+00}$	2.932×10^{-02}		0.959	5.797
SCLJ	1024		1.0		0.9	0.8500	-3.408	$3.002\times10^{+00}$	4.276×10^{-02}		0.967	5.670
SCLJ	1024		1.0		1.1	0.8500	-3.209	$2.741\times10^{+00}$	5.855×10^{-02}		0.973	5.570
SCLJ	1024		1.0		1.3	0.8500	-3.050	$2.573\times10^{+00}$	7.058×10^{-02}		0.977	5.489
SCLJ	1024		1.0		1.5	0.8500	-2.919	$2.476\times10^{+00}$	8.529×10^{-02}		0.979	5.422
SCLJ	1024		1.0		1.7	0.8500	-2.809	$2.403\times10^{+00}$	1.000×10^{-01}		0.981	5.363
SCLJ	1024		1.0		1.9	0.8500	-2.715	$2.350\times10^{+00}$	1.126×10^{-01}		0.983	5.314

Continued on next page

Supplementary Table 1 Simulations for potentials in the “Lennard-Jones” unit system based upon the length scale and energy scale of the A particle, R is the U-W correlation, and γ is the density-scaling exponent

pot.	N_{A}	N_{B}	m_{A}	m_{B}	T	ρ	$S_{\mathrm{ex}}/(Nk_{\mathrm{B}})$	η	D_{A}	D_{B}	R	γ
SCLJ	1024		1.0		2.1	0.8500	-2.632	$2.321\times10^{+00}$	1.276×10^{-01}		0.984	5.269
SCLJ	1024		1.0		3	0.8500	-2.358	$2.231\times10^{+00}$	1.908×10^{-01}		0.988	5.121
SCLJ	1024		1.0		4	0.8500	-2.159	$2.219\times10^{+00}$	2.501×10^{-01}		0.991	5.011
SCLJ	1024		1.0		5	0.8500	-2.018	$2.227\times10^{+00}$	3.207×10^{-01}		0.992	4.932
SCLJ	1024		1.0		7	0.8500	-1.825	$2.292\times10^{+00}$	4.346×10^{-01}		0.994	4.822
SCLJ	1024		1.0		8	0.8500	-1.754	$2.337\times10^{+00}$	4.800×10^{-01}		0.995	4.781
SCLJ	1024		1.0		10	0.8500	-1.644	$2.423\times10^{+00}$	5.825×10^{-01}		0.995	4.717
WS	512	512	2.0	1.0	0.67	1.2960	-5.247	$1.464\times10^{+02}$	1.100×10^{-03}	1.654×10^{-03}	0.983	5.085
WS	512	512	2.0	1.0	0.75	1.2960	-5.017	$5.497\times10^{+01}$	2.441×10^{-03}	3.432×10^{-03}	0.985	5.120
WS	512	512	2.0	1.0	0.849	1.2960	-4.792	$3.096\times10^{+01}$	4.502×10^{-03}	6.317×10^{-03}	0.987	5.130
WS	512	512	2.0	1.0	1.037	1.2960	-4.460	$1.801\times10^{+01}$	8.993×10^{-03}	1.234×10^{-02}	0.989	5.112
WS	512	512	2.0	1.0	1.226	1.2960	-4.207	$1.340\times10^{+01}$	1.356×10^{-02}	1.877×10^{-02}	0.990	5.080
WS	512	512	2.0	1.0	1.414	1.2960	-4.003	$1.113\times10^{+01}$	1.875×10^{-02}	2.527×10^{-02}	0.991	5.050
WS	512	512	2.0	1.0	1.603	1.2960	-3.835	$9.712\times10^{+00}$	2.465×10^{-02}	3.189×10^{-02}	0.992	5.021
WS	512	512	2.0	1.0	1.791	1.2960	-3.692	$8.827\times10^{+00}$	2.902×10^{-02}	3.922×10^{-02}	0.992	4.995
WS	512	512	2.0	1.0	1.98	1.2960	-3.568	$8.196\times10^{+00}$	3.572×10^{-02}	4.769×10^{-02}	0.993	4.970
WS	512	512	2.0	1.0	3	1.2960	-3.105	$6.573\times10^{+00}$	6.687×10^{-02}	8.537×10^{-02}	0.994	4.870
WS	668	334	2.0	1.0	0.4	1.1000	-5.412	$1.707\times10^{+02}$	6.415×10^{-04}	8.744×10^{-04}	0.951	5.242
WS	668	334	2.0	1.0	0.5	1.1000	-4.978	$3.288\times10^{+01}$	2.630×10^{-03}	3.713×10^{-03}	0.967	5.455
WS	668	334	2.0	1.0	0.6	1.1000	-4.671	$1.800\times10^{+01}$	5.250×10^{-03}	7.264×10^{-03}	0.973	5.461
WS	668	334	2.0	1.0	0.7	1.1000	-4.432	$1.302\times10^{+01}$	8.268×10^{-03}	1.146×10^{-02}	0.977	5.432
WS	668	334	2.0	1.0	0.8	1.1000	-4.236	$1.061\times10^{+01}$	1.145×10^{-02}	1.503×10^{-02}	0.979	5.396
WS	668	334	2.0	1.0	1	1.1000	-3.929	$8.181\times10^{+00}$	1.775×10^{-02}	2.347×10^{-02}	0.983	5.328
WS	668	334	2.0	1.0	1.2	1.1000	-3.697	$7.006\times10^{+00}$	2.451×10^{-02}	3.421×10^{-02}	0.985	5.268
WS	668	334	2.0	1.0	1.4	1.1000	-3.513	$6.349\times10^{+00}$	3.320×10^{-02}	4.252×10^{-02}	0.986	5.218
WS	668	334	2.0	1.0	2	1.1000	-3.126	$5.408\times10^{+00}$	5.450×10^{-02}	7.097×10^{-02}	0.989	5.102
WS	750	250	2.0	1.0	0.4	1.1000	-5.824	$5.253\times10^{+03}$	5.979×10^{-05}	1.021×10^{-04}	0.967	5.334
WS	750	250	2.0	1.0	0.415	1.1000	-5.739	$1.613\times10^{+03}$	1.266×10^{-04}	2.024×10^{-04}	0.970	5.300
WS	750	250	2.0	1.0	0.435	1.1000	-5.643	$6.641\times10^{+02}$	2.411×10^{-04}	3.748×10^{-04}	0.972	5.264
WS	750	250	2.0	1.0	0.45	1.1000	-5.574	$3.690\times10^{+02}$	3.720×10^{-04}	5.417×10^{-04}	0.974	5.341
WS	750	250	2.0	1.0	0.5	1.1000	-5.368	$1.269\times10^{+02}$	9.548×10^{-04}	1.356×10^{-03}	0.978	5.350
WS	750	250	2.0	1.0	0.6	1.1000	-5.039	$3.951\times10^{+01}$	2.685×10^{-03}	3.773×10^{-03}	0.981	5.346
WS	750	250	2.0	1.0	0.8	1.1000	-4.564	$1.734\times10^{+01}$	7.407×10^{-03}	1.016×10^{-02}	0.985	5.292
WS	750	250	2.0	1.0	1	1.1000	-4.229	$1.207\times10^{+01}$	1.290×10^{-02}	1.659×10^{-02}	0.987	5.233
WS	750	250	2.0	1.0	1.2	1.1000	-3.974	$9.583\times10^{+00}$	1.855×10^{-02}	2.491×10^{-02}	0.989	5.181
WS	750	250	2.0	1.0	1.4	1.1000	-3.772	$8.276\times10^{+00}$	2.540×10^{-02}	3.309×10^{-02}	0.990	5.136
WS	750	250	2.0	1.0	2	1.1000	-3.348	$6.517\times10^{+00}$	4.577×10^{-02}	5.989×10^{-02}	0.992	5.035
WS	750	250	2.0	1.0	2.7	1.5000	-5.057	$1.144\times10^{+02}$	4.672×10^{-03}	6.872×10^{-03}	0.998	4.562
WS	750	250	2.0	1.0	3	1.5000	-4.870	$7.463\times10^{+01}$	7.600×10^{-03}	1.004×10^{-02}	0.998	4.553
WS	750	250	2.0	1.0	4	1.5000	-4.392	$3.685\times10^{+01}$	1.865×10^{-02}	2.579×10^{-02}	0.999	4.527
WS	750	250	2.0	1.0	8	1.5000	-3.430	$1.695\times10^{+01}$	7.534×10^{-02}	9.965×10^{-02}	0.999	4.456
WS	750	250	2.0	1.0	12	1.5000	-2.979	$1.388\times10^{+01}$	1.389×10^{-01}	1.761×10^{-01}	0.999	4.413
WS	750	250	2.0	1.0	10	2.0000	-4.978	$2.200\times10^{+02}$	9.663×10^{-03}	1.346×10^{-02}	0.999	4.281
WS	750	250	2.0	1.0	12	2.0000	-4.657	$1.209\times10^{+02}$	1.974×10^{-02}	2.646×10^{-02}	0.999	4.273
WS	750	250	2.0	1.0	14	2.0000	-4.402	$8.556\times10^{+01}$	3.065×10^{-02}	4.229×10^{-02}	0.999	4.266
WS	750	250	2.0	1.0	18	2.0000	-4.016	$5.827\times10^{+01}$	5.658×10^{-02}	7.803×10^{-02}	0.999	4.254
WS	750	250	2.0	1.0	20	2.0000	-3.865	$5.144\times10^{+01}$	6.974×10^{-02}	9.062×10^{-02}	0.999	4.249
WS	820	204	2.0	1.0	0.5	1.0000	-4.689	$1.616\times10^{+01}$	4.948×10^{-03}	6.710×10^{-03}	0.965	5.669
WS	820	204	2.0	1.0	0.6	1.0000	-4.412	$1.156\times10^{+01}$	8.098×10^{-03}	1.087×10^{-02}	0.970	5.609
WS	820	204	2.0	1.0	0.7	1.0000	-4.193	$9.103\times10^{+00}$	1.142×10^{-02}	1.548×10^{-02}	0.974	5.553
WS	820	204	2.0	1.0	0.8	1.0000	-4.011	$7.802\times10^{+00}$	1.494×10^{-02}	2.033×10^{-02}	0.977	5.502
WS	820	204	2.0	1.0	1	1.0000	-3.727	$6.379\times10^{+00}$	2.312×10^{-02}	2.954×10^{-02}	0.980	5.417
WS	820	204	2.0	1.0	1.2	1.0000	-3.511	$5.648\times10^{+00}$	3.092×10^{-02}	3.896×10^{-02}	0.983	5.346
WS	820	204	2.0	1.0	1.6	1.0000	-3.199	$4.880\times10^{+00}$	4.675×10^{-02}	6.355×10^{-02}	0.986	5.240
WS	820	204	2.0	1.0	2	1.0000	-2.979	$4.480\times10^{+00}$	6.349×10^{-02}	8.154×10^{-02}	0.988	5.158

Supplementary Table 2 Simulations for CuZr mixtures with the length scale l_0 of Å, mass dimension m_0 of unit (1 u = 1.6605390666 × 10^{-27} kg), and energy scale e_0 of eV, R is the U-W correlation, and γ is the density-scaling exponent

pot.	N_{Cu}	N_{Zr}	m_{Cu} / u	m_{Zr} / u	T / K	ρ / Å^{-3}	$S_{\mathrm{ex}}/(Nk_{\mathrm{B}})$	η / $l_0^2/\sqrt{e_0 m_0}$	D_{Cu} / $l_0\sqrt{e_0/m_0}$	D_{Zr} / $l_0\sqrt{e_0/m_0}$	R	γ
CuZr	360	640	63.546	91.224	4000	0.0800	-3.901	$5.865\times10^{+00}$	4.291×10^{-03}	3.661×10^{-03}	0.912	1.856
CuZr	360	640	63.546	91.224	3000	0.0800	-4.477	$1.131\times10^{+01}$	1.765×10^{-03}	1.444×10^{-03}	0.903	1.892
CuZr	360	640	63.546	91.224	2500	0.0800	-4.880	$2.376\times10^{+01}$	7.553×10^{-04}	6.303×10^{-04}	0.897	1.912
CuZr	360	640	63.546	91.224	2200	0.0800	-5.182	$6.019\times10^{+01}$	3.040×10^{-04}	2.514×10^{-04}	0.893	1.925
CuZr	360	640	63.546	91.224	2000	0.0800	-5.418	$2.246\times10^{+02}$	1.079×10^{-04}	8.443×10^{-05}	0.890	1.936
CuZr	360	640	63.546	91.224	1900	0.0800	-5.549	$7.261\times10^{+02}$	4.778×10^{-05}	3.764×10^{-05}	0.889	1.945
CuZr	360	640	63.546	91.224	1850	0.0800	-5.618	$1.810\times10^{+03}$	2.712×10^{-05}	2.227×10^{-05}	0.890	1.954
CuZr	360	640	63.546	91.224	1800	0.0800	-5.690	$5.456\times10^{+03}$	1.496×10^{-05}	1.086×10^{-05}	0.889	1.956
CuZr	640	360	63.546	91.224	4000	0.0800	-3.362	$3.169\times10^{+00}$	7.334×10^{-03}	5.924×10^{-03}	0.955	2.394
CuZr	640	360	63.546	91.224	3000	0.0800	-3.854	$4.680\times10^{+00}$	3.805×10^{-03}	3.131×10^{-03}	0.953	2.464
CuZr	640	360	63.546	91.224	2400	0.0800	-4.287	$7.610\times10^{+00}$	2.008×10^{-03}	1.549×10^{-03}	0.952	2.512
CuZr	640	360	63.546	91.224	2000	0.0800	-4.679	$1.475\times10^{+01}$	9.123×10^{-04}	7.152×10^{-04}	0.951	2.548
CuZr	640	360	63.546	91.224	1800	0.0800	-4.925	$2.813\times10^{+01}$	5.125×10^{-04}	3.708×10^{-04}	0.950	2.568
CuZr	640	360	63.546	91.224	1600	0.0800	-5.221	$9.640\times10^{+01}$	1.802×10^{-04}	1.297×10^{-04}	0.952	2.594
CuZr	640	360	63.546	91.224	1500	0.0800	-5.397	$2.472\times10^{+02}$	7.365×10^{-05}	4.577×10^{-05}	0.954	2.621
