# Supplementary material for: Excess-entropy scaling in supercooled binary mixtures
Source: Nat Commun. 2020 Aug 27;11:4300. doi: 10.1038/s41467-020-17948-1 (PMC7453028; doi:10.1038/s41467-020-17948-1)
Supplement: Supplementary file 3 — Description of Additional Supplementary Files [file 41467_2020_17948_MOESM3_ESM.pdf]

Description of Additional Supplementary Files

File Name: Supplementary Data 1

Description: csv data for binary mixtures using the Lennard-Jones unit system.

File Name: Supplementary Data 2

Description: csv data for binary metallic alloys.
